# Heart diseases and echocardiography in rural Tanzania: Occurrence, characteristics, and etiologies of underappreciated cardiac pathologies

**DOI:** 10.1371/journal.pone.0208931

**Published:** 2018-12-26

**Authors:** Dominick M. Raphael, Laurine Roos, Victor Myovela, Elisante Mchomvu, Jabir Namamba, Said Kilindimo, Winfrid Gingo, Christoph Hatz, Daniel H. Paris, Maja Weisser, Richard Kobza, Martin Rohacek

**Affiliations:** 1 St. Francis Referral Hospital, Ifakara, United Republic of Tanzania; 2 University of Basel, Basel, Switzerland; 3 Emergency Department, Muhimbili University of Health and Allied Sciences, Dar es Salaam, United Republic of Tanzania; 4 Swiss Tropical and Public Health Institute, Basel, Switzerland; 5 Ifakara Health Institute, Ifakara, United Republic of Tanzania; 6 Division of Infectious Diseases, University Hospital Basel, Basel, Switzerland; 7 Division of Cardiology, Luzerner Kantonsspital, Luzern, Switzerland; Ziekenhuisgroep Twente, NETHERLANDS

## Abstract

**Background:**

Little is known about heart diseases and their treatment in rural sub-Saharan Africa. This study aimed to describe the occurrence, characteristics, and etiologies of heart diseases, and the medication taken before and prescribed after echocardiography in a rural referral Hospital in Tanzania.

**Methods:**

This prospective descriptive cohort study included all adults and children referred for echocardiography. Clinical and echocardiographic data were collated for analysis.

**Results:**

From December 2015 to October 2017, a total of 1’243 echocardiograms were performed. A total of 815 adults and 59 children ≤15 years had abnormal echocardiographic findings; in adults 537/815 (66%) had hypertension, with 230/537(43%) on antihypertensive drugs, and 506/815 (62%) were not on regular cardiac medication; 346/815 (42%) had severe eccentric or concentric left ventricular hypertrophy, and 182/815 (22%) had severe systolic heart failure. Only 44% demonstrated normal left ventricular systolic function. The most frequent heart diseases were hypertensive heart disease (41%), valvular heart disease (18%), coronary heart disease (18%), peripartum cardiomyopathy (7%), and other non-hypertensive dilated cardiomyopathies (6%) in adults, and congenital heart disease (34%) in children. Following echocardiography, 802/815 (98%) adults and 40/59 (68%) children had an indication for cardiac medication, 70/815 (9%) and 2/59 (3%) for oral anticoagulation, and 35/815 (4%) and 23/59 (39%) for cardiac surgery, respectively.

**Conclusion:**

Hypertension is the leading etiology of heart diseases in rural Tanzania. Most patients present with advanced stages of heart disease, and the majority are not treated before echocardiography. There is an urgent need for increased awareness, expertise and infrastructure to detect and treat hypertension and heart failure in rural Africa.

## Introduction

Non-communicable diseases are underappreciated in countries with a low-middle socio-demographic index (SDI), even though disability adjusted life-years (DALYs, the sum of the years of life lost due to premature mortality and years of life lived with disability) due to non-communicable diseases increased by 54% during the last 25 years [1].

Although DALYs from communicable, maternal, and nutritional diseases still account for more than those from non-communicable diseases in sub-Saharan Africa, cardiovascular diseases are increasingly present among the leading causes of total DALYs in countries with a low SDI [1]. According to World Health Organisation (WHO) estimates, cardiovascular diseases are the second most common cause of death in Africa. In 2015, almost 1.2 million people died because of cardiovascular diseases in Africa, which is more than for malaria and tuberculosis combined [2]. Mortality of patients with heart failure has been reported to be highest in Africa, compared to other low- and middle income regions: The INTER CF prospective cohort study including patients from Asia, South America, Africa, and the Middle East showed a one year overall mortality of 16.5%, but for Africa alone the mortality was estimated at 34% [3]. The most commonly reported etiology of heart failure in low- and middle-income regions is ischemic heart disease, except for the Americas and Africa, where hypertension is the predominant cause [4]. A recent systematic review and meta-analysis of 22 studies from Africa (1999–2017) including 10’098 patients found hypertensive heart disease (39.2%) to be the commonest cause of heart failure, followed by cardiomyopathies (21.4%), and rheumatic heart disease (14.1%). Ischemic heart disease was reported to be rare (7.2%) [5]. However, most patients included were from urban hospitals, and only a minority (603/10’098, 6%) originated from rural areas, where cardiomyopathies and rheumatic heart disease appeared to be most common causes for heart failure in small studies from rural Rwanda and rural Cameroon [6, 7]. In the same review [5], 9 included studies analyzed the use of cardiac medication given at admission or at discharge from a hospital. However, only 11% of the patients represented rural areas from sub-Saharan Africa, and the proportion of patients on cardiac medication at presentation to a clinic was not described yet. In this study, we describe the occurrence, characteristics, and etiologies of heart diseases diagnosed using echocardiography in patients from the rural Kilombero- and Ulanga districts in Tanzania, and describe the medication taken before and prescribed after echocardiography.

## Material and methods

### Ethics statement

The study was approved by ethics committee in Switzerland (Ethikkommission Nordwest und Zentralschweiz (EKNZ UBE-15/83)) and the ethics committees of the Ifakara Health Institute (Institutional Review Board, IHI/IRB/No 38–2015) as well as the National Institute for Medical Research, Tanzania (Ref. NIMR/HQ/R.8a/Vol. IX/2242). All three committees waived informed consent, to minimize selection bias, and due to the substantial benefit for the study population.

### Study site

The St Francis Referral Hospital is situated in Ifakara in Tanzania and serves as a referral center for a rural population of about one million inhabitants form the Kilombero- and Ulanga districts. It has 360 beds and specialized services in internal medicine, surgery, obstetrics, neonatology and gynecology, ophthalmology, pediatrics, and has an HIV and tuberculosis clinic. In September 2015, an emergency department including a triaging system and training of medical staff in emergency medicine, sonography and echocardiography was implemented. Since January 2016, this emergency department has been open 24 hours a day, caring for over 36’000 patients per year, and is the only site in the Kilombero- and Ulanga region where echocardiography is available.

### Study design

All symptomatic patients (i.e adults including pregnant women, and children ≤15 years with dyspnea, orthopnoea or chest pain of all ages) who were referred to the emergency department for echocardiography were eligible for this prospective descriptive cohort study. Over 22 consecutive months the medical histories, clinical examination results and echocardiographic data were collected for these patients and collated for analysis.

### Data collection

Clinical data (medical history including symptoms and medication, physical examination including weight, height, body surface area, blood pressure (BP) and heart rate) were consecutively collected on a dedicated case report form (EpiData). BP was measured with a manual BP meter in sitting position, after a minimum of 5 minutes of rest. Prior to echocardiography, history taking and physical examination including inspection, palpation of the apex beat, and auscultation of the heart and lungs was performed in all patients by the echocardiographer. Initial electrocardiograms (ECG) and chest x-rays were done according to the decision of the referring clinician.

### Echocardiography

Two-dimensional echocardiography with color-, pulse wave (PW)-, continuous wave (CW)- and tissue doppler imaging (TDI) was performed with a Mindray M7 ultrasound machine (Mindray, Shenzhen, China) using a P4-2s phased array transducer. Detailed echocardiographic assessment of ventricular and atrial size, left- and right ventricular systolic and diastolic function, regional wall abnormalities, and valvular structures and function was conducted and interpreted according to recent recommendations and international guidelines [8–15]. End-systolic and end-diastolic left ventricular (LV) volumes and LV ejection fraction (EF) were calculated by the Simpson method of discs. To measure and interpret LV diastolic function and to estimate LV end-diastolic- and left atrial filling pressure (LAP), mitral inflow- (E/A), mitral septal annulus- (e’), and tricuspid regurgitation (TR) velocity, E/e’, left atrium (LA) volume index, and pulmonary venous flow was used. In case of valvular heart disease with mitral valve stenosis or at least moderate mitral valve regurgitation, diastolic function and LAP was considered to be determinable only if Ar-A≥30msec, and in case of depressed EF only, if septal E/e’ >15 [9]. To measure the right ventricular function, tricuspid annular plane systolic excursion (TAPSE), right ventricular systolic excursion velocity by TDI (s’), fractional area change (FAC), and myocardial performance index (MPI) was used. To estimate pulmonary artery pressure, TR velocity, MPI, pulmonary valve acceleration time/right ventricular ejection time (PVAccT/RVET), and deceleration index (DI) was used. The right atrial pressure was estimated by inferior vena cava (IVC) diameter and the presence of its inspiratory collapse. To evaluate the severity of valvular regurgitation, an integrative approach was used, including the proximal isovelocity surface area (PISA) method if appropriate [10]. To quantify mitral stenosis, planimetry of the valve area and mean gradient was used. All echocardiographic procedures were done by the last author, who prescribed all medication, and recommended heart surgery and anticoagulation according to current guidelines [16–20].

### Definitions

#### Hypertension

Systolic BP >140mmHg or diastolic BP >90mmHg, measured after a minimum of 5 minutes of rest in sitting position, and at least one documented BP of >140/90mmHg at another occasion, or presence of an established antihypertensive therapy and a documented BP of >140/90mmHg in the past [21, 22].

#### Hypertensive heart disease

Hypertrophy or concentric remodeling of the left ventricle with or without global systolic or diastolic left ventricular dysfunction in a patient with arterial hypertension, with neither valve disease nor segmental wall motion abnormalities.

#### Dilated cardiopathy

Eccentric hypertrophy of the left ventricle and dilated atria or dilated right ventricle.

#### Valvular heart disease

Abnormal size and/or function of the heart and a primary abnormality of a valve (i.e. presence of valve regurgitation or stenosis and thickening of cusps, leaflets, or leaflet tips, vegetations or ruptured chordae tendineae).

#### Rheumatic heart disease

Definition according to the 2012 world heart federation (WHF) criteria for echocardiographic diagnosis of rheumatic heart disease [23].

#### Endocarditis

Definition according the Duke criteria for diagnosis of infective endocarditis [24].

#### Coronary heart disease

Typical angina pectoris and ventricular dysfunction with segmental hypo- or akinesia which could be attributed to a specific coronary artery with or without typical ECG-findings.

#### Peripartum cardiomyopathy

Cardiomyopathy with a reduced left ventricular ejection fraction of <45%, presenting towards the end of the pregnancy or in the months after delivery in a woman without previously known structural heart disease [25].

#### Hypertrophic cardiomyopathy

A heart with a LV with concentric hypertrophy.

#### Right heart failure

Right ventricular dysfunction with a fractional area change (FAC) <32%.

#### Pulmonary heart disease

Right heart failure in presence of pulmonary hypertension and normal LAP.

#### TB-pericarditis

Pericardial effusion in a patient with clinically suspected or microbiologically confirmed tuberculosis (TB).

#### Constrictive pericarditis

Echocardiographical signs of constriction in a patient with suspected or confirmed TB.

#### Endomyocardial fibrosis and congenital heart diseases

Definition according to current recommendations [19, 26].

#### Hypertrophy of the left ventricle in children

LV mass (LVM)/height^2.7^ above the 95^th^ percentile [13]; severe LV hypertrophy: LVM of at least 30% above the 95^th^ percentile.

#### Concentric hypertrophy

Increased LVM and a relative wall thickness (RWT) ≥0.42; **eccentric hypertrophy,** increased LVM and RWT <0.42; **concentric remodelling**, normal LVM with a RWT ≥0.42 [8].

### Statistical analysis

In this study only descriptive statistical analysis was applied, using Microsoft Excel, after export of the data from EpiData.

## Results

From December 2015 through to October 2017, a total of 1’243 echocardiograms were performed. After exclusion of 278 (22%) echocardiograms of patients with completely normal findings and 64 (5%) echocardiograms with non-relevant findings only (i.e minimal or mild mitral valve regurgitation in presence of normal leaflet morphology, and no other evidence of heart disease), 901 echocardiograms of symptomatic patients with a heart disease were included. After exclusion of 27 follow-up examinations, 874 echocardiograms of 874 patients were analyzed.

### Patient characteristics

Table 1 outlines the patient’s characteristics: A total of 815 were adults, and 59 were children ≤15 years. A total of 199/874 (23%) were admitted to the hospital ward. Median age of the adults was 59 years (range 16–98), 329/815 (40%) were male, 282/815 (35%) had overweight or were obese, and 537/815 (66%) had hypertension. A total of 41/815 (5%) adults were HIV positive. At presentation, 506/815 (62%) were not on regular medication. Among those who had hypertension, only 230/537 (43%) took at least one antihypertensive drug, while BP was >140/90mmHg in 358/537 (67%), and BP was ≥160/100mmHg in 287/537 (53%). A total of 68/815 (8.3%) had atrial fibrillation.

**Table 1:** Baseline characteristics.


Patient characteristics	Adults (n = 815)	Children (n = 59)
Age [years], median (range)	59 (16–98)	5 (0.003–15)
Children <5 years, n (%)	N/A	28 (47%)
Male sex, n (%)	329 (40%)	27 (46%)
Weight [kg] (n = 793)/(n = 55), median (range)	60 (30–150)	16 (2–43)
Height [cm] (n = 793)/(n = 51), median (range)	160 (103–190)	106 (50–162)
BSA [m^2^] (n = 793)/(n = 51), median (range)	1.62 (1.1–2.66)	0.7 (0.22–1.41)
BMI [kg/m^2^] (n = 793)/(n = 51), median (range)	23.19 (13.16–57.85)	N/A
Underweight (n = 793)/(n = 51), n (%)	86 (11%)	17 (33%)
Normal weight (n = 793)/(n = 51), n (%)	425 (54%)	28 (55%)
Overweight (n = 793)/(n = 51), n (%)	165 (21%)	5 (10%)
Obesity (n = 793)/(n = 51), n (%)	117 (15%)	1 (2%)
Hypertension, n (%)	537 (66%)	2 (3%)
Stroke, n (%)	53 (7%)	0 (0%)
Pregnancy in the last 6 months, n (%)	68 (8%)	0 (0%)
No medication before presentation, n (%)	506 (62%)	44 (75%)
Inpatient, n (%)	178 (22%)	21 (36%)
HIV positive, n (%)	41 (5%)	0 (0%)
**Symptoms and clinical findings**		
Dyspnoea, n (%)	485 (60%)	32 (54%)
Orthopnoea, n (%)	398 (49%)	19 (32%)
Chest pain, n (%)	230 (28%)	8 (14%)
Systolic BP [mmHg] (n = 806)/(n = 23), median (range)	140 (70–260)	115 (80–165)
Diastolic BP [mmHg] (n = 806)/(n = 23), median (range)	80 (30–150)	70 (40–120)
HR [beat/min] (n = 764)/(n = 44), median (range)	80 (36–160)	110 (70–200)
Irregular heartbeat, n (%)	118 (14%)	3 (5%)
Dilated jugular veins, n (%)	217 (27%)	2 (3%)
Apical beat lateralised, n (%)	257 (32%)	15 (25%)
Heart murmur, n (%)	158 (19%)	31 (53%)

N/A, not applicable; BSA, body surface area; BMI, body mass index; HR, heart rate; BP, blood pressure.

The numbers (n = )/(n = ) after each patient characteristic indicate for how many adults and children data were available.

Underweight: BMI <18.5 kg/m^2^ for adults, or BMI for age 2 or 3 standard deviations (SD) below the WHO growth reference for children ≥5 years old, or weight for height 2 or 3 SD below the WHO growth reference for children <5 years old; Normal weight: BMI 18.5 to <25 kg/m^2^ for adults, or children who have no overweight nor obesity nor underweight; Overweight: BMI 25 to <30 kg/m^2^ for adults, or BMI for age 1 SD above the WHO growth reference for children ≥5 years old, or weight for height 2 SD above the WHO growth reference for children <5 years old; Obesity: BMI ≥30 kg/m^2^ for adults, or BMI for age ≥2 SD above the WHO growth reference for children ≥5 years old, or weight for height 3 SD above the WHO growth reference for children <5 years old.

The median age of children presenting was 5 years (range 0.003–15), of which 27/59 (46%) were male, and 17/59 (33%) had underweight.

### Echocardiographic findings

Table 2 outlines interpretations of echocardiographic findings, which are shown in detail in S1 Table. Only 93/815 (11%) adults had a normal sized left ventricle, and 353/815 (44%) had a normal left ventricular systolic function. On the other hand, 346/815 (42%) had severe eccentric or concentric hypertrophy of the left ventricle, and 182/815 (22%) had severe systolic heart failure.

**Table 2:** Interpretation of echocardiographic findings.

		
	Adults (n = 815)	Children (n = 59)
Normal LV size, n (%)	93 (11%)	29 (49%)
Concentric remodelling, n (%)	161 (20%)	10 (17%)
Concentric hypertrophy, n (%)	299 (37%)	6 (10%)
- Concentric hypertrophy, severe, n (%)	191 (23%)	5 (8%)
Eccentric hypertrophy, n (%)	262 (32%)	14 (24%)
- Eccentric hypertrophy, severe, n (%)	155 (19%)	10 (17%)
Normal systolic LV function (LVEF ≥54%) (n = 811)/(n = 58), n (%)	353 (44%)	44 (76%)
Mild systolic heart failure (LVEF 41–53%) (n = 811)/(n = 58), n (%)	101 (12%)	3 (5%)
Moderate systolic heart failure (LVEF 30–40%) (n = 811)/(n = 58), n (%)	175 (22%)	5 (9%)
Severe systolic heart failure (LVEF <30%) (n = 811)/(n = 58), n (%)	182 (22%)	6 (10%)
Diastolic relaxation impairment of left ventricle		
- None, n (%)	260 (32%)	25 (42%)
- Grade 1, n (%)	125 (15%)	2 (3%)
- Grade 2, n (%)	112 (14%)	5 (8%)
- Grade 3, n (%)	179 (22%)	7 (11%)
- Diastolic function not determinable, n (%)	139 (17%)	20 (33%)
Diastolic relaxation impairment grade 2 or 3 and LVEF ≥50%, n (%)	32 (4%)	1 (2%)
Right ventricle, size		
- Normal size, n (%)	444 (54%)	29 (49%)
- Dilated or hypertrophic, n (%)	371 (46%)	30 (51%)
Right ventricle, function		
- normal, n (%)	542 (67%)	42 (71%)
- impaired, n (%)	273 (33%)	17 (29%)
Pulmonary hypertension		
- no (n = 797)/(n = 58), n (%)	464 (58%)	34 (59%)
- due to left sided heart disease (i.e. elevated LAP) (n = 797)/(n = 58), n (%)	292 (37%)	17 (29%)
- due to other cause (n = 797)/(n = 58), n (%)	41 (5%)	7 (12%)
Aortic regurgitation, moderate or severe, n (%)	58 (7%)	1 (2%)
Aortic stenosis, moderate or severe, n (%)	3 (0%)	0 (0%)
Mitral regurgitation, moderate or severe, n (%)	157 (19%)	11 (19%)
Mitral stenosis, any, n (%)	16 (2%)	2 (3%)
Mitral stenosis, moderate or severe, n (%)	13 (2%)	2 (3%)
Mitral stenosis, severe, n (%)	8 (1%)	1 (2%)
Pulmonal regurgitation, moderate or severe, n (%)	11 (1%)	1 (2%)
Pulmonal stenosis, moderate or severe, n (%)	0 (0%)	6 (10%)
Tricuspid regurgitation, moderate or severe, n (%)	101 (12%)	11 (19%)

LV, left ventricle; LVEF, left ventricular ejection fraction; LAP, left atrial pressure. The numbers (n = )/(n = ) indicate for how many adults and children data were available.

### Etiology of heart diseases

**Fig 1:**
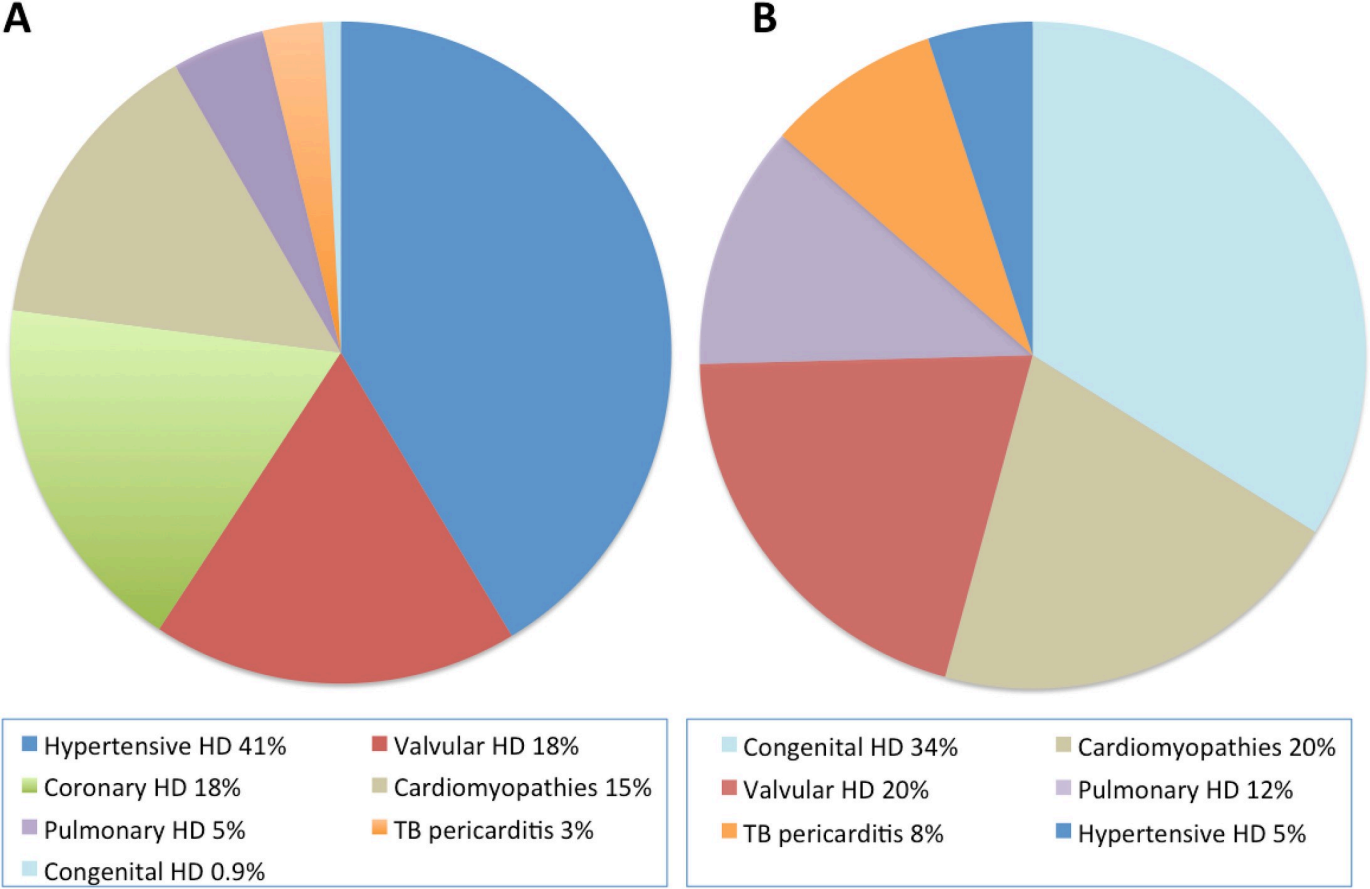
**Distribution of heart diseases in adults (A) and in children (B).** HD, heart disease; TB, tuberculosis.

Hypertension was the main cause or a concomitant cause for heart disease in 510/815 (63%) adults. Table 3 and Fig 1 gives an overview of the distribution of different heart diseases in adults and children. In adults, the most frequent heart diseases were hypertensive heart disease (337/815, 41%), valvular heart disease (146/815, 18%), coronary heart disease (145/815, 18%), peripartum cardiomyopathy (56/815, 7%), other non-hypertensive dilated cardiomyopathies (52/815, 6%), and pulmonary heart disease (37/815, 5%).

**Table 3:** Causes of heart disease in 874 patients.

		
	Adults (n = 815)	Children (n = 59)
Hypertensive HD, n (%)	337 (41%)	3 (5%)
- Hypertensive dilated cardiopathy with eccentric hypertrophy, n (%)	76 (9%)	1 (2%)
- Hypertensive HD with concentric hypertrophy, n (%)	160 (20%)	2 (3%)
Valvular HD, n (%)	146 (18%)	12 (20%)
- Rheumatic HD, n (%)	68 (8%)	5 (8%)
- Endocarditis, n (%)	9 (1%)	2 (3%)
- Valvular HD with hypertension, n (%)	43 (5%)	0 (0%)
Coronary HD, n (%)	145 (18%)	0 (0%)
- Coronary HD without hypertension, n (%)	29 (4%)	0 (0%)
- Coronary HD with hypertension, n (%)	116 (14%)	0 (0%)
Peripartum cardiomyopathy, n (%)	56 (7%)	0 (0%)
- Peripartum cardiomyopathy with hypertension, n (%)	14 (2%)	0 (0%)
Dilated cardiomyopathy, other, n (%)	52 (6%)	9 (15%)
Hypertrophic cardiomyopathy, other, n (%)	8 (1%)	2 (3%)
Endomyocardial fibrosis, n (%)	3 (0.4%)	1 (2%)
TB-pericarditis, n (%)	16 (2%)	5 (8%)
Pericarditis constrictiva, n (%)	8 (1%)	0 (0%)
Right heart failure due to pulmonal hypertension/normal LAP, n (%)	37 (5%)	7 (12%)
Arrhythmogenic right ventricular cardiomyopathy, n (%)	2 (0.2%)	1 (2%)
VSD*, n (%)	2 (0.2%)	6 (10%)
ASD, n (%)	3 (0.4%)	6 (10%)
TOF, n (%)	0 (0%)	6 (10%)
Complete AV defect, n (%)	0 (0%)	1 (2%)
PDA, n (%)	0 (0%)	6 (10%)

HD, heart disease; TB, tuberculosis; LAP, left atrial pressure; VSD, ventricular septal defect; ASD, atrial septal defect; TOF, Tetralogy of Fallot; AV, atrioventricular; PDA, patent ductus arteriosus. * VSD in absence of TOF. Children with congenital heart disease: n = 20 (34%). Two children with TOF had concomitant ASD and one child with TOF had concomitant PDA. One child had VSD and ASD, one child had VSD, ASD and PDA.

S2 Table outlines detailed echocardiographic findings of adult patients with rheumatic heart disease. Of the 146 adults with valvular heart disease, 68/146 (47%) fulfilled the criteria for rheumatic heart disease according to WHF criteria [23]. Most common was moderate or severe mitral regurgitation, which was present in 40/68 (59%) patients. Out of 16 patients with mitral stenosis, 13 (81%) were moderate or severe.

A total of 48/68 (71%) patients had an impaired left ventricular systolic function, and 32/68 (47%) had an impaired right ventricular function.

S2 Table outlines detailed echocardiographic findings of patients with peripartum cardiomyopathy. Of 56 patients with peripartum cardiomyopathy, 48/56 (86%) had moderate or severe systolic heart failure, and 21/56 (38%) had severe eccentric hypertrophy.

Heart failure with preserved ejection fraction (HFpEF): Of all patients, only 32/815 (4%) had HFpEF (i.e. EF ≥ 50% and elevated LV-filling pressure).

The most frequent heart diseases in children were congenital heart disease (20/59, 34%), valvular heart disease (12/59, 20%), with 5/12 (42%) fulfilling the criteria for rheumatic heart disease, dilated cardiomyopathy (9/59, 15%), and right heart failure due to pulmonal hypertension (7/59, 12%).

### Indications for medication and cardiac surgery

After echocardiography, 802/815 (98%) adults and 40/59 (68%) children had an indication for cardiac medication, 70/815 (9%) adults and 2/59 (3%) children for oral anticoagulation, and 35/815 (4%) adults and 23/59 (39%) children for cardiac surgery. In 700/815 (86%) adults and 32/59 (54%) children at least one new drug was added, in 199/815 (24%) adults and 6/59 (10%) children the dose of at least one drug was increased, and in 191/815 (23%) adults and 9/59 (15%) children, at least one drug was stopped.

**Fig 2:**
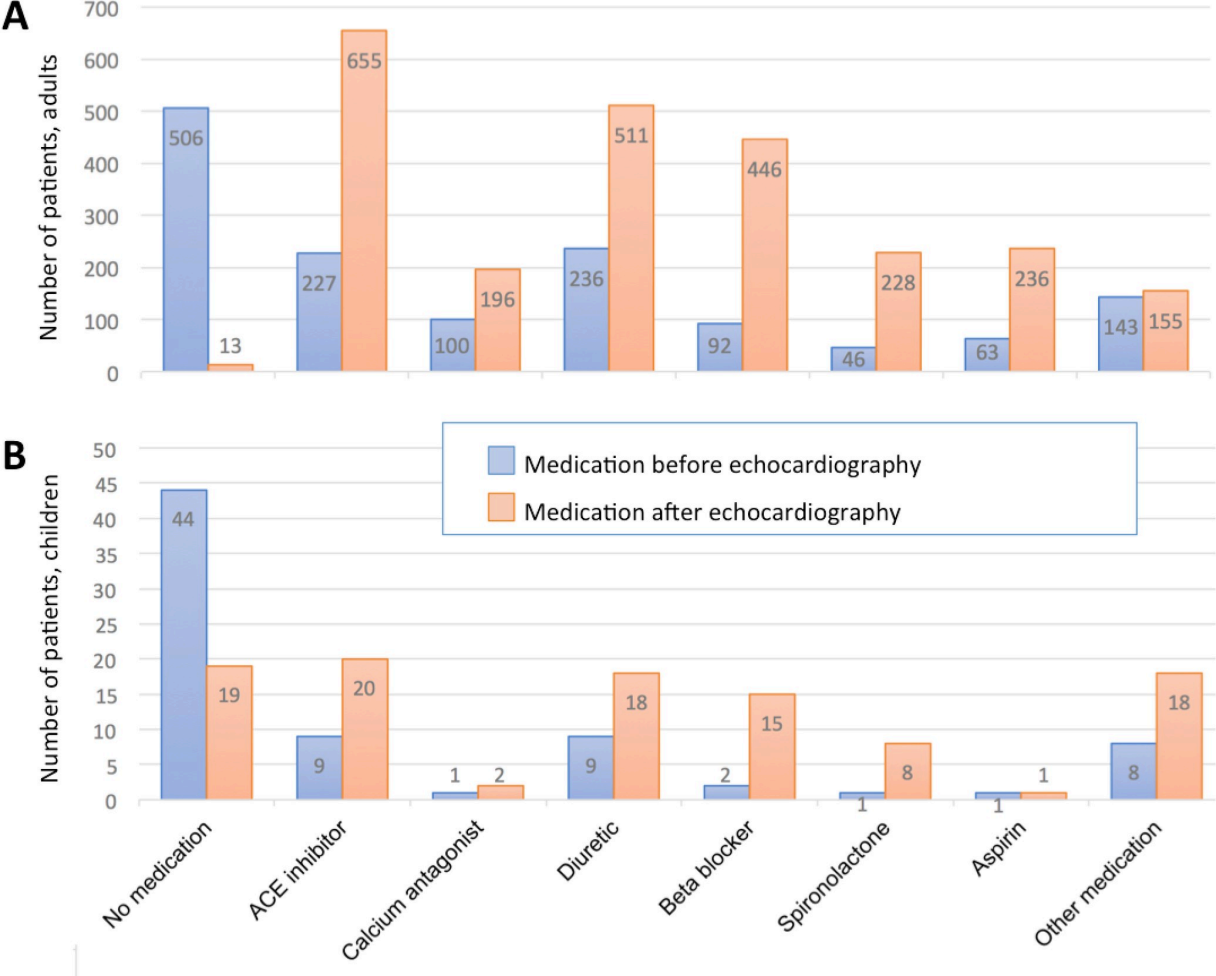
**Medication taken before and prescribed after echocardiography in adults (A) and in children (B)**.

Fig 2 shows the drugs taken before and prescribed after echocardiography. Most prescribed drugs after echocardiography were ACE-inhibitors, diuretics, and beta-blockers.

## Discussion

Of 1’243 echocardiograms performed within 22 consecutive months, 901 (72%) had relevant pathologies. Most patients presented with advanced stages of heart failure. The majority of patients with heart disease had not been treated before echocardiography, although almost all had an indication for treatment. The most frequent heart disease was hypertensive heart disease, followed by valvular-, and coronary heart disease, and cardiomyopathies. More than one third of children had an indication for cardiac surgery, and 9% of the adults for oral anticoagulation. High blood pressure was not well controlled in two thirds of the patients with hypertension. To date, this is the largest prospective cohort study reporting on occurrence, characteristics, and etiologies of heart diseases conducted at a rural site in Africa—our results reflect the extent and burden of heart diseases, and the neglect of their awareness and management in sub-Saharan Africa.

The high frequency of hypertensive heart disease is in line with the recent meta-analysis of 22 African studies in mostly urban patients [5], and stands in contrast to two studies from rural Rwanda and Cameroon, where cardiomyopathies and rheumatic heart disease were more common [6, 7]. We observed more coronary heart disease, less rheumatic heart disease, and less cardiomyopathies than previously reported [5]. However, it is likely that the prevalence of coronary heart disease was underestimated in previous studies, due to inadequate diagnostic methods, a paucity of physicians, and unreliable health statistics [27]. The high occurrence of poorly controlled hypertension, and the high proportion of untreated patients at presentation in our study is in line with a recent large cross-sectional study, which showed a high cumulative prevalence of cardiovascular risk factors in urban and rural Malawi. The crude prevalence of hypertension was 14.7% in the urban area and 13.6% in the rural area, and many people with hypertension (900/2208 (41%) urban and 1188 /1888 (63%) rural) were not overweight or obese. Only 29% were taking regular antihypertensive medication, and less than half of those had a well-controlled BP (i.e. below 140/90mmHg) [28]. A meta-analysis of studies from 2000 to 2013 in sub-Saharan Africa showed that the prevalence of hypertension at ages of 30, 40, 50 and 60 years was 16%, 26%, 35%, and 44%, respectively. Only 7%-56% were aware of their hypertensive status, 18% were receiving antihypertensive drugs, but only 7% had adequately controlled blood pressure [29]. In another observational cross-sectional study in mostly urban areas of 12 sub-Saharan African countries, 77% had uncontrolled hypertension, although 97% were under antihypertensive medication. The proportion of uncontrolled hypertension increased with decreasing level of patient wealth, and higher grade of hypertension was seen in the lower wealth categories [30]. Among other risk factors such as overweight and reduced physical activity, a reduced urine sodium concentrating ability, increased sodium sensitivity, low plasma renin values, mutations of epithelial sodium channels and other genetic factors might contribute to hypertension in Africans [31–33]. Although Africans did not consume more salt than Whites or Indians in a south-African study, they consumed more than 6 grams of salt per day, which is more than recommended by the WHO [34].

In this study, patients presented with advanced stages of heart failure, notably patients with peripartum cardiomyopathy and rheumatic heart disease. In patients with peripartum cardiomyopathy, severe heart failure and increased left ventricular end-diastolic diameter is associated with non-improvement of left ventricular function [35], and in patients with rheumatic heart disease, it is too late for cardiac surgery in case of severe heart failure [17].

We observed only 32 (4%) cases of heart failure with preserved ejection fraction (HFpEF), defined as heart failure with EF≥50% and a conclusively measurable diastolic relaxation impairment of grade 2 or 3 (i.e. elevated left ventricular filling pressure), following recent recommendations [9]. Studies from the USA and Europe indicate that up to 50% of patients with heart failure have preserved ejection fraction [36], and two studies from sub-Saharan Africa and one study including Latin American, middle-eastern, and North African regions reported proportions of 53%-65% of HFpEF in their study populations [37–39]. In most studies including those from Africa, the definition of HFpEF was based on clinical signs and symptoms of heart failure and preserved left ventricular EF, and diastolic function was not reported. If we would use a similar definition of HFpEF, we would count 405 (50%) cases with HFpEF, and if the same echocardiographic criteria would be used as in an older American prospective study, 27% of our patients would have had isolated diastolic dysfunction and HFpEF, compared to 44% in that study [40].

In accordance to the high prevalence of not treated hypertension, the majority of our patients were not treated for heart failure upon presentation–but after echocardiography, patients were not only prescribed additional drugs, but unnecessary drugs were stopped, or doses of established drugs adapted. Moreover, indication for oral anticoagulation and surgery was made. Most prescribed drugs after performed echocardiography were ACE-Inhibitors, diuretics, beta-blockers, and spironolactone. All of these drugs have an impact on morbidity and mortality: ACE-inhibitors, beta-blockers and spironolactone reduce death and hospitalization by about one third each, the latter in patients with severe heart failure only [41]. Before, information on proportions of treated patients with heart failure was lacking for African regions. All studies reporting on medication described drugs given at admission or at discharge from a hospital, or the time of prescription was not defined [5].

Following echocardiography, 9% of the adult patients had an indication for oral anticoagulation. However, to measure international normalized ratio (INR) was not possible in our setting. Direct anticoagulants such as Rivaroxaban are available, but they are not affordable by most of the patients. Thus, we could not anticoagulate patients with atrial fibrillation. Data about the use of anticoagulants in sub-Saharan Africa except from south-Africa are scarce [42]. A survey among cardiologists in Cameroon showed that only one third of patients with an indication for anticoagulation because of atrial fibrillation received anticoagulation [43]. One study done in 6 tertiary hospitals in Nigeria found suboptimal usage of heparin or warfarin to be common, with reduced awareness of direct anticoagulants among residents and consultants [44].

One third of the children had an indication for cardiac surgery. While the outcome of congenital heart diseases such as Tetralogy of Fallot is poor without an intervention (only one fourth reach the age of 10 years)[45], it is excellent after surgical repair [46], and the outcome after mitral valve replacement in children is good, especially for older children [47]. However, given the ratio of one cardiac center per 33.3 to 50.5 million people and only 1.6 open heart operations per million people per year in sub-Saharan Africa [48], patients face a challenge to get cardiac surgery, also because of unaffordably high costs.

The proportion of people living with HIV was 5% in our study population, which corresponds to the national HIV prevalence of 5%[49]. One third of the children had underweight, which is more than the prevalence of underweight in children 0 to 59 months (13.4% (95% CI 12.7–14.1) in the mainland of Tanzania [50]. Underweight in children with heart disease is common, and depends on multiple factors such as severity of cardiac disease, increased energy requirements, decreased nutritional intake, malabsorbtion, and poor utilisation of absorbed nutrition [51].

This study has several limitations: First, we could not follow-up all patients—thus we cannot comment on long-term outcomes and survival rates or adherence to the prescribed medication. Meanwhile, (V.M) was trained in echocardiography, and clinical and echocardiographic follow-up is done. Future trials performed at our site would allow to build a cardiology center offering systematic follow-up and anticoagulation. Second, the diagnosis of coronary heart disease was based on history, clinical examination, electrocardiogram, and echocardiographic findings. In this rural setting, we could neither perform coronary angiography nor myocardial scintigraphy nor stress-echocardiography. Thus, coronary heart disease remained a suspected diagnosis. Third, we did not screen for cardiovascular risk factors other than hypertension and overweight. One cross-sectional study including 9134 people living in Ifakara showed that 5% had diabetes and 30% had hypertension. A total of 17% of men and 2% of women did smoke, and 15% of men and 6% of women consumed alcohol. Only 8% led sedentary lives [52]. Forth, we could not perform myocardial biopsies, and histological determination of the origin of cardiomyopathies was not possible. Finally, existing echocardiographic normograms for chamber size have not been validated for the African population.

This study has several strengths: The sample size is relatively large, compared to other studies; all patients referred for echocardiography were included prospectively, and patients were examined regardless of their wealth status or availability of health insurance. Selection bias could be minimized, as echocardiography is available only at our site for two large rural districts of Tanzania, and virtually all patients referred for echocardiography from these districts were included. We were able to present detailed echocardiographic measurements and interpretations according to the latest recommendations, and medications were prescribed and surgery recommended following current international guidelines.

In conclusion, hypertension is extremely common and mostly not controlled in rural Tanzania. The leading cause for heart disease in this setting is hypertension. Most patients presented with advanced stages of heart diseases, and the majority was not treated for heart failure before echocardiography. There is an urgent need for community education to improve awareness and to prevent hypertension, for trained staff, and for infrastructure to manage hypertension and heart failure in rural Africa.

## Supporting information

S1 Table Detailed echocardiographic findings of all patients. LVEDD: left ventricular end-diastolic diameter; IVSd: intraventricular septum in diastole; PWd: posterior wall in diastole; LV: left ventricle; RWT: relative wall thickness; LVOT: left ventricular outflow tract; LA: right atrium; LAVI: left atrial volume index; RA: right atrium; EF: ejection fraction; TAPSE: tricuspid annular plane systolic excursion; FAC: fractional area change; MV: mitral valve; DT: deceleration time; TR: tricuspid regurgitation; Pvein: pulmonary vein. N/A, not applicable. The numbers (n = )/(n = ) indicate of how many adults and children, data were available. * Cantinotti et al 2014 [14].
(DOCX)

S2 Table Echocardiographic findings in adults (n = 815) with rheumatic heart disease and peripartum cardiomyopathy. LVEDD: left ventricular end-diastolic diameter; IVSd: intraventricular septum in diastole; PWd: posterior wall in diastole; LV: left ventricle; RWT: relative wall thickness; LVOT: left ventricular outflow tract; LA: right atrium; LAVI: left atrial volume index; RA: right atrium; EF: ejection fraction; TAPSE: tricuspid annular plane systolic excursion; FAC: fractional area change; MV: mitral valve; DT: deceleration time; TR: tricuspid regurgitation; Pvein: pulmonary vein. The numbers (n = )/(n = ) indicate for how many adults and children, data were available.
(DOCX)

S3 Table Dataset adults. (XLSX)

S4 Table Dataset, children. (XLSX)

## References

[1] DALYs GBD, Collaborators H. Global, regional, and national disability-adjusted life-years (DALYs) for 333 diseases and injuries and healthy life expectancy (HALE) for 195 countries and territories, 1990–2016: a systematic analysis for the Global Burden of Disease Study 2016. Lancet. 2017;390(10100):1260–344. 10.1016/S0140-6736(17)32130-X 28919118 PMC5605707

[2] WHO. Global Health Estimates 2015: Death by Cause, Age, Sex, by Country and by Region, 2000–2015 Geneva, World Health Organization 2016.

[3] Dokainish H, Teo K, Zhu J, Roy A, AlHabib KF, ElSayed A, et al Global mortality variations in patients with heart failure: results from the International Congestive Heart Failure (INTER-CHF) prospective cohort study. Lancet Glob Health. 2017;5(7):e665–e72. 10.1016/S2214-109X(17)30196-1 . 28476564

[4] Callender T, Woodward M, Roth G, Farzadfar F, Lemarie JC, Gicquel S, et al Heart failure care in low- and middle-income countries: a systematic review and meta-analysis. PLoS Med. 2014;11(8):e1001699 10.1371/journal.pmed.1001699 25117081 PMC4130667

[5] Agbor VN, Essouma M, Ntusi NAB, Nyaga UF, Bigna JJ, Noubiap JJ. Heart failure in sub-Saharan Africa: A contemporaneous systematic review and meta-analysis. Int J Cardiol. 2018;257:207–15. 10.1016/j.ijcard.2017.12.048 . 29506693

[6] Kwan GF, Bukhman AK, Miller AC, Ngoga G, Mucumbitsi J, Bavuma C, et al A simplified echocardiographic strategy for heart failure diagnosis and management within an integrated noncommunicable disease clinic at district hospital level for sub-Saharan Africa. JACC Heart Fail. 2013;1(3):230–6. 10.1016/j.jchf.2013.03.006 . 24621875

[7] Tantchou Tchoumi JC, Ambassa JC, Kingue S, Giamberti A, Cirri S, Frigiola A, et al Occurrence, aetiology and challenges in the management of congestive heart failure in sub-Saharan Africa: experience of the Cardiac Centre in Shisong, Cameroon. Pan Afr Med J. 2011;8:11 22121420 10.4314/pamj.v8i1.71059 PMC3201578

[8] Lang RM, Badano LP, Mor-Avi V, Afilalo J, Armstrong A, Ernande L, et al Recommendations for cardiac chamber quantification by echocardiography in adults: an update from the American Society of Echocardiography and the European Association of Cardiovascular Imaging. Eur Heart J Cardiovasc Imaging. 2015;16(3):233–70. 10.1093/ehjci/jev014 . 25712077

[9] Nagueh SF, Smiseth OA, Appleton CP, Byrd BF 3rd, Dokainish H, Edvardsen T, et al Recommendations for the Evaluation of Left Ventricular Diastolic Function by Echocardiography: An Update from the American Society of Echocardiography and the European Association of Cardiovascular Imaging. Eur Heart J Cardiovasc Imaging. 2016;17(12):1321–60. 10.1093/ehjci/jew082 . 27422899

[10] Zoghbi WA, Enriquez-Sarano M, Foster E, Grayburn PA, Kraft CD, Levine RA, et al Recommendations for evaluation of the severity of native valvular regurgitation with two-dimensional and Doppler echocardiography. J Am Soc Echocardiogr. 2003;16(7):777–802. 10.1016/S0894-7317(03)00335-3 . 12835667

[11] Baumgartner H, Hung J, Bermejo J, Chambers JB, Evangelista A, Griffin BP, et al Echocardiographic assessment of valve stenosis: EAE/ASE recommendations for clinical practice. J Am Soc Echocardiogr. 2009;22(1):1–23; quiz 101–2. 10.1016/j.echo.2008.11.029 . 19130998

[12] Rudski LG, Lai WW, Afilalo J, Hua L, Handschumacher MD, Chandrasekaran K, et al Guidelines for the echocardiographic assessment of the right heart in adults: a report from the American Society of Echocardiography endorsed by the European Association of Echocardiography, a registered branch of the European Society of Cardiology, and the Canadian Society of Echocardiography. J Am Soc Echocardiogr. 2010;23(7):685–713; quiz 86–8. 10.1016/j.echo.2010.05.010 . 20620859

[13] Khoury PR, Mitsnefes M, Daniels SR, Kimball TR. Age-specific reference intervals for indexed left ventricular mass in children. J Am Soc Echocardiogr. 2009;22(6):709–14. 10.1016/j.echo.2009.03.003 . 19423289

[14] Cantinotti M, Scalese M, Murzi B, Assanta N, Spadoni I, De Lucia V, et al Echocardiographic nomograms for chamber diameters and areas in Caucasian children. J Am Soc Echocardiogr. 2014;27(12):1279–92 e2. 10.1016/j.echo.2014.08.005 . 25240494

[15] Lopez L, Colan SD, Frommelt PC, Ensing GJ, Kendall K, Younoszai AK, et al Recommendations for quantification methods during the performance of a pediatric echocardiogram: a report from the Pediatric Measurements Writing Group of the American Society of Echocardiography Pediatric and Congenital Heart Disease Council. J Am Soc Echocardiogr. 2010;23(5):465–95; quiz 576–7. 10.1016/j.echo.2010.03.019 . 20451803

[16] Yancy CW, Jessup M, Bozkurt B, Butler J, Casey DE Jr., Drazner MH, et al 2013 ACCF/AHA guideline for the management of heart failure: a report of the American College of Cardiology Foundation/American Heart Association Task Force on Practice Guidelines. J Am Coll Cardiol. 2013;62(16):e147–239. 10.1016/j.jacc.2013.05.019 . 23747642

[17] Nishimura RA, Otto CM, Bonow RO, Carabello BA, Erwin JP 3rd, Guyton RA, et al 2014 AHA/ACC guideline for the management of patients with valvular heart disease: executive summary: a report of the American College of Cardiology/American Heart Association Task Force on Practice Guidelines. J Am Coll Cardiol. 2014;63(22):2438–88. 10.1016/j.jacc.2014.02.537 . 24603192

[18] Minette MS, Sahn DJ. Ventricular septal defects. Circulation. 2006;114(20):2190–7. 10.1161/CIRCULATIONAHA.106.618124 . 17101870

[19] Villafane J, Feinstein JA, Jenkins KJ, Vincent RN, Walsh EP, Dubin AM, et al Hot topics in tetralogy of Fallot. J Am Coll Cardiol. 2013;62(23):2155–66. 10.1016/j.jacc.2013.07.100 . 24076489

[20] January CT, Wann LS, Alpert JS, Calkins H, Cigarroa JE, Cleveland JC Jr., et al 2014 AHA/ACC/HRS guideline for the management of patients with atrial fibrillation: a report of the American College of Cardiology/American Heart Association Task Force on Practice Guidelines and the Heart Rhythm Society. J Am Coll Cardiol. 2014;64(21):e1–76. 10.1016/j.jacc.2014.03.022 . 24685669

[21] Messerli FH, Williams B, Ritz E. Essential hypertension. Lancet. 2007;370(9587):591–603. 10.1016/S0140-6736(07)61299-9 . 17707755

[22] Dannenberg AL, Garrison RJ, Kannel WB. Incidence of hypertension in the Framingham Study. Am J Public Health. 1988;78(6):676–9. 3259405 10.2105/ajph.78.6.676 PMC1350281

[23] Remenyi B, Wilson N, Steer A, Ferreira B, Kado J, Kumar K, et al World Heart Federation criteria for echocardiographic diagnosis of rheumatic heart disease—an evidence-based guideline. Nat Rev Cardiol. 2012;9(5):297–309. 10.1038/nrcardio.2012.7 22371105 PMC5523449

[24] Durack DT, Lukes AS, Bright DK. New criteria for diagnosis of infective endocarditis: utilization of specific echocardiographic findings. Duke Endocarditis Service. Am J Med. 1994;96(3):200–9. . 8154507 10.1016/0002-9343(94)90143-0

[25] Sliwa K, Hilfiker-Kleiner D, Petrie MC, Mebazaa A, Pieske B, Buchmann E, et al Current state of knowledge on aetiology, diagnosis, management, and therapy of peripartum cardiomyopathy: a position statement from the Heart Failure Association of the European Society of Cardiology Working Group on peripartum cardiomyopathy. Eur J Heart Fail. 2010;12(8):767–78. 10.1093/eurjhf/hfq120 . 20675664

[26] Grimaldi A, Mocumbi AO, Freers J, Lachaud M, Mirabel M, Ferreira B, et al Tropical Endomyocardial Fibrosis: Natural History, Challenges, and Perspectives. Circulation. 2016;133(24):2503–15. 10.1161/CIRCULATIONAHA.115.021178 . 27297343

[27] Onen CL. Epidemiology of ischaemic heart disease in sub-Saharan Africa. Cardiovasc J Afr. 2013;24(2):34–42. 10.5830/CVJA-2012-071 23612951 PMC3734874

[28] Price AJ, Crampin AC, Amberbir A, Kayuni-Chihana N, Musicha C, Tafatatha T, et al Prevalence of obesity, hypertension, and diabetes, and cascade of care in sub-Saharan Africa: a cross-sectional, population-based study in rural and urban Malawi. Lancet Diabetes Endocrinol. 2018;6(3):208–22. 10.1016/S2213-8587(17)30432-1 29371076 PMC5835666

[29] Ataklte F, Erqou S, Kaptoge S, Taye B, Echouffo-Tcheugui JB, Kengne AP. Burden of undiagnosed hypertension in sub-saharan Africa: a systematic review and meta-analysis. Hypertension. 2015;65(2):291–8. 10.1161/HYPERTENSIONAHA.114.04394 . 25385758

[30] Antignac M, Diop IB, Macquart de Terline D, Kramoh KE, Balde DM, Dzudie A, et al Socioeconomic Status and Hypertension Control in Sub-Saharan Africa: The Multination EIGHT Study (Evaluation of Hypertension in Sub-Saharan Africa). Hypertension. 2018;71(4):577–84. 10.1161/HYPERTENSIONAHA.117.10512 . 29378852

[31] Garfinkle MA. Salt and essential hypertension: pathophysiology and implications for treatment. J Am Soc Hypertens. 2017;11(6):385–91. 10.1016/j.jash.2017.04.006 . 28479261

[32] Yako YY, Balti EV, Matsha TE, Dzudie A, Kruger D, Sobngwi E, et al Genetic factors contributing to hypertension in African-based populations: A systematic review and meta-analysis. J Clin Hypertens (Greenwich). 2018;20(3):485–95. 10.1111/jch.13225 . 29520984 PMC8031059

[33] Opie LH, Seedat YK. Hypertension in sub-Saharan African populations. Circulation. 2005;112(23):3562–8. 10.1161/CIRCULATIONAHA.105.539569 . 16330697

[34] Swanepoel B, Schutte AE, Cockeran M, Steyn K, Wentzel-Viljoen E. Sodium and potassium intake in South Africa: an evaluation of 24-hour urine collections in a white, black, and Indian population. J Am Soc Hypertens. 2016;10(11):829–37. 10.1016/j.jash.2016.08.007 . 27720143

[35] Haghikia A, Podewski E, Libhaber E, Labidi S, Fischer D, Roentgen P, et al Phenotyping and outcome on contemporary management in a German cohort of patients with peripartum cardiomyopathy. Basic Res Cardiol. 2013;108(4):366 10.1007/s00395-013-0366-9 23812247 PMC3709080

[36] Dunlay SM, Roger VL, Redfield MM. Epidemiology of heart failure with preserved ejection fraction. Nat Rev Cardiol. 2017;14(10):591–602. 10.1038/nrcardio.2017.65 . 28492288

[37] Abebe TB, Gebreyohannes EA, Tefera YG, Abegaz TM. Patients with HFpEF and HFrEF have different clinical characteristics but similar prognosis: a retrospective cohort study. BMC Cardiovasc Disord. 2016;16(1):232 10.1186/s12872-016-0418-9 27871223 PMC5117494

[38] Bonsu KO, Owusu IK, Buabeng KO, Reidpath DD, Kadirvelu A. Clinical characteristics and prognosis of patients admitted for heart failure: A 5-year retrospective study of African patients. Int J Cardiol. 2017;238:128–35. 10.1016/j.ijcard.2017.03.014 . 28318656

[39] Magana-Serrano JA, Almahmeed W, Gomez E, Al-Shamiri M, Adgar D, Sosner P, et al Prevalence of heart failure with preserved ejection fraction in Latin American, Middle Eastern, and North African Regions in the I PREFER study (Identification of Patients With Heart Failure and PREserved Systolic Function: an epidemiological regional study). Am J Cardiol. 2011;108(9):1289–96. 10.1016/j.amjcard.2011.06.044 . 22000627

[40] Bursi F, Weston SA, Redfield MM, Jacobsen SJ, Pakhomov S, Nkomo VT, et al Systolic and diastolic heart failure in the community. JAMA. 2006;296(18):2209–16. 10.1001/jama.296.18.2209 . 17090767

[41] Ponikowski P, Voors AA, Anker SD, Bueno H, Cleland JGF, Coats AJS, et al 2016 ESC Guidelines for the diagnosis and treatment of acute and chronic heart failure: The Task Force for the diagnosis and treatment of acute and chronic heart failure of the European Society of Cardiology (ESC)Developed with the special contribution of the Heart Failure Association (HFA) of the ESC. Eur Heart J. 2016;37(27):2129–200. 10.1093/eurheartj/ehw128 . 27206819

[42] Mazurek M, Huisman MV, Rothman KJ, Paquette M, Teutsch C, Diener HC, et al Regional Differences in Antithrombotic Treatment for Atrial Fibrillation: Insights from the GLORIA-AF Phase II Registry. Thromb Haemost. 2017;117(12):2376–88. 10.1160/TH17-08-0555 . 29212125 PMC6260111

[43] Ntep-Gweth M, Zimmermann M, Meiltz A, Kingue S, Ndobo P, Urban P, et al Atrial fibrillation in Africa: clinical characteristics, prognosis, and adherence to guidelines in Cameroon. Europace. 2010;12(4):482–7. 10.1093/europace/euq006 . 20179174

[44] Anakwue R, Nwagha T, Ukpabi OJ, Obeka N, Onwubuya E, Onwuchekwa U, et al Clinicians-related determinants of anticoagulation therapy and prophylaxis in Nigeria. Ann Afr Med. 2017;16(4):164–9. 10.4103/aam.aam_35_17 29063899 PMC5676405

[45] Bertranou EG, Blackstone EH, Hazelrig JB, Turner ME, Kirklin JW. Life expectancy without surgery in tetralogy of Fallot. Am J Cardiol. 1978;42(3):458–66. . 685856 10.1016/0002-9149(78)90941-4

[46] Tennant PW, Pearce MS, Bythell M, Rankin J. 20-year survival of children born with congenital anomalies: a population-based study. Lancet. 2010;375(9715):649–56. 10.1016/S0140-6736(09)61922-X . 20092884

[47] Henaine R, Roubertie F, Vergnat M, Ninet J. Valve replacement in children: a challenge for a whole life. Arch Cardiovasc Dis. 2012;105(10):517–28. 10.1016/j.acvd.2012.02.013 . 23062483

[48] Yankah C, Fynn-Thompson F, Antunes M, Edwin F, Yuko-Jowi C, Mendis S, et al Cardiac surgery capacity in sub-saharan Africa: quo vadis? Thorac Cardiovasc Surg. 2014;62(5):393–401. 10.1055/s-0034-1383723 . 24955755

[49] Ministry of Health, United Republic of Tanzania. Tanzania HIV Impact Survey 2016–2017. http://wwwnbsgotz/nbs/takwimu/this2016-17/Tanzania_SummarySheet_Englishpdf. 2017.

[50] Ministry of Health and Welfare, United Republic of Tanzania. Tanzania National Nutrition Survey 2014 https://wwwuniceforg/tanzania/Tanzania_National_Nutrition_Survey_2014_Final_Report_18012015pdf. 2014.

[51] Argent AC, Balachandran R, Vaidyanathan B, Khan A, Kumar RK. Management of undernutrition and failure to thrive in children with congenital heart disease in low- and middle-income countries. Cardiol Young. 2017;27(S6):S22–S30. 10.1017/S104795111700258X . 29198259

[52] Ramaiya A, Geubbels E. HIV and NCD: The burden of chronic disease in Rural Tanzania. Spotlight. 2014;https://drive.google.com/file/d/0B7y5ETfTjl4xREwyQUg0SkgzR0k/edit(19).

