# Supplementary material for: Heart diseases and echocardiography in rural Tanzania: Occurrence, characteristics, and etiologies of underappreciated cardiac pathologies
Source: PLoS One. 2018 Dec 26;13(12):e0208931. doi: 10.1371/journal.pone.0208931 (PMC6306243; doi:10.1371/journal.pone.0208931)
Supplement: S1 Table — LVEDD: left ventricular end-diastolic diameter; IVSd: intraventricular septum in diastole; PWd: posterior wall in diastole; LV: left ventricle; RWT: relative wall thickness; LVOT: left ventricular outflow tract; LA: right atrium; LAVI: left atrial volume index; RA: right atrium; EF: ejection fraction; TAPSE: tricuspid annular plane systolic excursion; FAC: fractional area change; MV: mitral valve; DT: deceleration time; TR: tricuspid regurgitation; Pvein: pulmonary vein. N/A, not applicable. The numbers (n = )/(n = ) indicate of how many adults and children, data were available. * Cantinotti et al 2014 [14]. (DOCX) [file pone.0208931.s001.docx]

|  | | |
| --- | --- | --- |
|  | **Adults (n=815)** | **Children (n=59)** |
| **Dimensions and LV Function** | | |
| LVEDD [mm] (n=812)/(n=57), median (range) | 48.3 (15-108) | 34.8 (10.5-70.8) |
| IVSd [mm] (n=812)/(n=57), median (range) | 11.4 (4.4-28) | 6.5 (2-15) |
| PWd [mm] (n=813)/(n=57), median (range) | 10.9 (5.8-26) | 6.3 (2.2-14.5) |
| LV mass Index [g/m^2^] (n=789), median (range) | 125.53 (26-424.29) | N/A |
| LV mass index >95 in females, >115 in males (n=789), n (%) | 554 (70%) | N/A |
| RWT (n=812)/(n=57), median (range) | 0.46 (0.2-2.48) | 0.37 (0.18-1.04) |
| RWT >0.42 (n=812)/(n=57), n (%) | 475 (58%) | 22 (39%) |
| LVOT [mm] (n=781)/(n=52), median (range) | 18 (10-32) | 1.5 (3.8-18.8) |
| Aorta ascendens [mm] (n=737)/(n=33), median (range) | 27 (16-51.2) | 17.7 (5-26) |
| LA [mm] (n=789)/(n=54), median (range) | 38 (15-91) | 27 (9-62) |
| LA [ml] (n=790)/(n=53), median (range) | 50 (15-200) | 20 (2-90) |
| LAVI [ml/m^2^] (n=770)/(n=48), median (range) | 33.02 (10.29-136.07) | 34.08 (9.1-111) |
| LAVI > 34 [ml/m^2^] (n=771), n (%) | 368 (48%) | N/A |
| RA [ml] (n=777)/(n=48), median (range) | 44 (12-268) | 21.5 (2-106) |
| RA enlarged (>32ml/m^2^ in male, >27ml/m^2^ in female) (n=757), n (%) | 353 (47%) | N/A |
| V. cava [mm] (n=766)/(n=50), median (range) | 15.6 (1-36) | 10.35 (2-22) |
| V. cava >2cm (n=766)/(n=50), n (%) | 193 (25%) | 2 (4%) |
| V. cava collapsing >50% during inspiration, n (%) | 498 (61%) | 40 (68%) |
| LV ejection fraction [%] (n=811)/(n=58), median (range) | 48 (10-75) | 65 (20-75) |
| EF <55% (n=811)/(n=58), n (%) | 458 (56%) | 14 (24%) |
| EF <30% (n=811)/(n=58), n (%) | 182 (22%) | 6 (10%) |
| Regional wall motion assessment | | |
| normal (n=814)/(n=59), n (%) | 312 (38%) | 34 (58%) |
| Hypo - Akinesia (n=814)/(n=59), n (%) | 458 (56%) | 15 (25%) |
| D-sign (n=814)/(n=59), n (%) | 39 (5%) | 7 (12%) |
| **Right Ventricle** | | |
| diameter at the base [mm] (n=758)/(n=53), median (range) | 39.55 (21-78) | 28 (10.4-59) |
| diameter at the base [mm] >41mm (n=758)/Z-score>2* (n=53), n (%) | 330 (44%) | 17 (32%) |
| TAPSE [mm] (n=762)/(n=51), median (range) | 21 (5-45) | 20 (2.8-34.8) |
| Sa [cm/s] (n=724)/(n=46), median (range) | 11.8 (4-25) | 12.5 (5.4-17.4) |
| FAC <35% (n=298)/(n=21), n (%) | 237 (80%) | 14 (67%) |
| Pulmonal arterial pressure [mmHg] (n=456)/(n=26), median (range) | 45.0 (18-80) | 48.5 (19-98) |
| **Diastolic Function** | | |
| peak E-wave velocity (cm/sec) (n=796)/(n=48), median (range) | 74 (7-230) | 97 (37-185) |
| peak A-wave velocity (cm/sec) (n=738)/(n=46), median (range) | 59 (14-149) | 49 (21-117) |
| MV E/A ratio (n=737)/(n=46), median (range) | 1.17 (0.27-6.2) | 1.85 (0.49-6) |
| MV E/A ratio ≥2 (n=737)/(n=46), n (%) | 217 (29%) | 21 (46%) |
| MV DT (msec) (n=749)/(n=41), median (range) | 132 (30-444) | 96 (54-228) |
| septal e' <7cm/sec (n=779)/(n=49), n (%) | 607 (78%) | 16 (33%) |
| lateral e' <10cm/sec (n=642)/(n=34), n (%) | 541 (84%) | 15 (44%) |
| septal E/e' ratio >15 (n=776)/(n=47), n (%) | 244 (31%) | 12 (26%) |
| TR velocity >2.8m/s (n=550)/(n=39), n (%) | 283 (51%) | 29 (74%) |
| Ar-A >30ms (n=282)/(n=12), n (%) | 88 (31%) | 5 (42%) |
| Pvein S/D <1 (n=206)/(n=9), n (%) | 92 (45%) | 4 (44%) |
