# Supplementary material for: Heart diseases and echocardiography in rural Tanzania: Occurrence, characteristics, and etiologies of underappreciated cardiac pathologies
Source: PLoS One. 2018 Dec 26;13(12):e0208931. doi: 10.1371/journal.pone.0208931 (PMC6306243; doi:10.1371/journal.pone.0208931)
Supplement: S2 Table — LVEDD: left ventricular end-diastolic diameter; IVSd: intraventricular septum in diastole; PWd: posterior wall in diastole; LV: left ventricle; RWT: relative wall thickness; LVOT: left ventricular outflow tract; LA: right atrium; LAVI: left atrial volume index; RA: right atrium; EF: ejection fraction; TAPSE: tricuspid annular plane systolic excursion; FAC: fractional area change; MV: mitral valve; DT: deceleration time; TR: tricuspid regurgitation; Pvein: pulmonary vein. The numbers (n = )/(n = ) indicate for how many adults and children, data were available. (DOCX) [file pone.0208931.s002.docx]

|  | | |
| --- | --- | --- |
|  | **Rheumatic**  **heart disease**  **(n=68)** | **Peripartum cardiomyopathy**  **(n=56)** |
| **Dimensions and LV Function** | | |
| LVEDD [mm] (n=68)/(n=56), median (range) | 54.85 (28.5-77.2) | 56 (44.4-75.8) |
| IVSd [mm] (n=68)/(n=56), median (range) | 9.65 (6-20.4) | 8.85 (6.2-15.8) |
| PWd [mm] (n=68)/(n=56), median (range) | 9.65 (6-20) | 8.95 (6.6-13) |
| LV mass Index [g/m^2^] (n=66)/(n=54), median (range) | 137.49 (50.9-355.1) | 121.77 (76.6-227) |
| LV mass index >95 in females, >115 in males, (n=66)/(n=54), n (%) | 55 (83%) | 47 (87%) |
| RWT (n=68)/(n=56), median (range) | 0.35 (0.21-1.03) | 0.31 (0.22-0.52) |
| RWT >0.42 (n=68)/(n=56), n (%) | 20 (29%) | 4 (7%) |
| LVOT [mm] (n=67)/(n=55), median (range) | 16.7 (10-22) | 17 (12.7-24) |
| Aorta ascendens [mm] (n=59)/(n=50), median (range) | 24 (16-40) | 23.15 (17.3-32) |
| LA [mm] (n=68)/(n=56), median (range) | 48 (27-91) | 40.5 (27-56) |
| LA [ml] (n=68)/(n=56), median (range) | 91 (25-200) | 58.5 (21-180) |
| LAVI [ml/m^2^] (n=66)/(n=54), median (range) | 60 (13.65-132.34) | 35.81 (14.79-86) |
| LAVI > 34 [ml/m^2^] (n=66)/(n=54), n (%) | 56 (85%) | 35 (65%) |
| RA [ml] (n=65)/(n=56), median (range) | 55 (18-180) | 50 (21-119) |
| RA enlarged (>32ml/m^2^ in male, >27ml/m^2^ in female) (n=63)/(n=54), n (%) | 46 (73%) | 36 (67%) |
| V. cava [mm] (n=68)/(n=56), median (range) | 19 (5-30) | 17.5 (1.6-29) |
| V. cava >2cm (n=68)/(n=56), n (%) | 27 (40%) | 8 (14%) |
| V. cava collapsing >50% during inspiration, n (%) | 26 (38%) | 22 (39%) |
| LV ejection fraction [%] (n=68)/(n=56), median (range) | 40 (15-75) | 30 (15-56) |
| EF <55% (n=68))/(n=56), n (%) | 48 (71%) | 54 (96%) |
| EF <30% (n=68)/(n=56), n (%) | 17 (25%) | 22 (39%) |
| Regional wall motion assessment | | |
| normal (n=68)/(n=55), n (%) | 17 (25%) | 2 (4%) |
| Hypo - Akinesia (n=68)/(n=55), n (%) | 43 (63%) | 53 (96%) |
| D-sign (n=68)/(n=55), n (%) | 6 (9%) | 0 (0%) |
| **Right Ventricle** | | |
| Diameter at the base [mm] (n=66)/(n=56), median (range) | 42.5 (25-63) | 44 (29-56) |
| Diameter at the base [mm] >41mm (n=66)/(n=56), n (%) | 37 (56%) | 33 (59%) |
| TAPSE [mm] (n=64)/(n=56), median (range) | 21 (8-32) | 19 (9.6-40) |
| Sa [cm/s] (n=62)/(n=54), median (range) | 11.5 (5-20) | 10.95 (7.3-16.2) |
| FAC <35% (n=37)/(n=33), n (%) | 27 (73%) | 31 (94%) |
| Pulmonal arterial pressure [mmHg] (n=49)/(n=37), median (range) | 50 (21-70) | 45 (23-69) |
| **Diastolic Function** | | |
| peak E-wave velocity (cm/sec) (n=61)/(n=55), median (range) | 107 (7-230) | 89 (7-182) |
| peak A-wave velocity (cm/sec) (n=53)/(n=53), median (range) | 61 (24-149) | 35 (24-121) |
| MV E/A ratio (n=54)/(n=54), median (range) | 1.52 (0.54-4) | 2.56 (0.81-6.2) |
| MV E/A ratio ≥2 (n=54)/(n=54), n (%) | 18 (33%) | 38 (70%) |
| MV DT (msec) (n=57)/(n=55), median (range) | 126 (60-408) | 102 (49-174) |
| septal e' <7cm/sec (n=63)/(n=55), n (%) | 54 (86%) | 43 (78%) |
| lateral e' <10cm/sec (n=43)/(n=50), n (%) | 38 (88%) | 48 (96%) |
| septal E/e' ratio >15 (n=61)/(n=55), n (%) | 41 (67%) | 29 (53%) |
| TR velocity >2.8m/s (n=58)/(n=46), n (%) | 45 (78%) | 21 (46%) |
| Ar-A >30ms (n=13)/(n=22), n (%) | 8 (62%) | 15 (68%) |
| Pvein S/D <1 (n=13)/(n=19), n (%) | 10 (77%) | 17 (89%) |
| **Valves** |  |  |
| Aortic regurgitation, moderate or severe, n (%) | 20 (29%) | 1 (2%) |
| Aortic regurgitation, severe, n (%) | 2 (3%) | 0 (0%) |
| Aortic stenosis, moderate or severe, n (%) | 0 (0%) | 0 (0%) |
| Mitral regurgitation, moderate or severe, n (%) | 40 (59%) | 15 (27%) |
| Mitral regurgitation, severe, n (%) | 26 (38%) | 2 (4%) |
| Mitral stenosis, any, n (%) | 16 (24%) | 1 (2%) |
| Mitral stenosis, moderate or severe, n (%) | 13 (19%) | 1 (2%) |
| Mitral stenosis, severe, n (%) | 8 (12%) | 0 (0%) |
| Pulmonal regurgitation, moderate or severe, n (%) | 4 (6%) | 0 (0%) |
| Pulmonal stenosis, moderate or severe, n (%) | 0 (0%) | 0 (0%) |
| Tricuspid regurgitation, moderate or severe, n (%) | 16 (24%) | 5 (9%) |
| **Interpretation of echocardiographic findings** | | |
| Normal LV size, n (%) | 8 (12%) | 6 (11%) |
| Concentric remodelling, n (%) | 4 (6%) | 1 (2%) |
| Concentric hypertrophy, n (%) | 15 (22%) | 5 (9%) |
| - Concentric hypertrophy, severe, n (%) | 14 (21%) | 4 (7%) |
| Eccentric hypertrophy, n (%) | 41 (60%) | 44 (79%) |
| - Eccentric hypertrophy, severe, n (%) | 25 (37%) | 21 (38%) |
| Normal systolic LV function (LVEF ≥54%) (n=68)/(n=56), n (%) | 20 (29%) | 2 (4%) |
| Mild systolic heart failure (LVEF 41-53%) (n=68)/(n=56), n (%) | 9 (13%) | 6 (11%) |
| Moderate systolic heart failure (LVEF 30-40%) (n=68)/(n=56), n (%) | 22 (32%) | 26 (46%) |
| Severe systolic heart failure (LVEF <30%) (n=68)/(n=56), n (%) | 17 (25%) | 22 (39%) |
| Diastolic relaxation impairment | | |
| - None, n (%) | 2 (3%) | 2 (4%) |
| - Grade 1, n (%) | 4 (6%) | 7 (13%) |
| - Grade 2, n (%) | 17 (25%) | 6 (11%) |
| - Grade 3, n (%) | 12 (18%) | 37 (66%) |
| - Diastolic function not determinable, n (%) | 33 (49%) | 4 (7%) |
| Elevated LV-filling pressure (n=57)/(n=54), n (%) | 29 (42%) | 43 (77%) |
| Right ventricle size | | |
| - Normal size, n (%) | 27 (40%) | 18 (32%) |
| - dilated, n (%) | 41 (60%) | 38 (68%) |
| Right ventricle function | | |
| - normal, n (%) | 36 (53%) | 24 (43%) |
| - impaired, n (%) | 32 (47%) | 32 (57%) |
| Pulmonary hypertension | | |
| - none (n=68)/(n=55), n (%) | 17 (25%) | 23 (42%) |
| - caused by left sided heart disease (i.e. elevated LAP)   (n=68)/(n=55), n (%) | 50 (74%) | 32 (58%) |
| - due to other cause (n=68)/(n=55), n (%) | 1 (1%) | 0 (0%) |
